# Improving translatability of spinal cord injury research by including age as a demographic variable

**DOI:** 10.3389/fncel.2022.1017153

**Published:** 2022-11-17

**Authors:** Andrew N. Stewart, Linda A. T. Jones, John C. Gensel

**Affiliations:** ^1^Department of Physiology, College of Medicine, University of Kentucky, Lexington, KY, United States; ^2^Spinal Cord and Brain Injury Research Center, College of Medicine, University of Kentucky, Lexington, KY, United States; ^3^Center for Outcomes and Measurement, Jefferson College of Rehabilitation Sciences, Thomas Jefferson University, Philadelphia, PA, United States

**Keywords:** neurotrauma, pre-clinical research, secondary injury, translation, sex as a biological variable

## Abstract

Pre-clinical and clinical spinal cord injury (SCI) studies differ in study design, particularly in the demographic characteristics of the chosen population. In clinical study design, criteria such as such as motor scores, neurological level, and severity of injury are often key determinants for participant inclusion. Further, demographic variables in clinical trials often include individuals from a wide age range and typically include both sexes, albeit historically most cases of SCI occur in males. In contrast, pre-clinical SCI models predominately utilize young adult rodents and typically use only females. While it is often not feasible to power SCI clinical trials to test multi-variable designs such as contrasting different ages, recent pre-clinical findings in SCI animal models have emphasized the importance of considering age as a biological variable prior to human experiments. Emerging pre-clinical data have identified case examples of treatments that diverge in efficacy across different demographic variables and have elucidated several age-dependent effects in SCI. The extent to which these differing or diverging treatment responses manifest clinically can not only complicate statistical findings and trial interpretations but also may be predictive of worse outcomes in select clinical populations. This review highlights recent literature including age as a biological variable in pre-clinical studies and articulates the results with respect to implications for clinical trials. Based on emerging unpredictable treatment outcomes in older rodents, we argue for the importance of including age as a biological variable in pre-clinical animal models prior to clinical testing. We believe that careful analyses of how age interacts with SCI treatments and pathophysiology will help guide clinical trial design and may improve both the safety and outcomes of such important efforts.

## Introduction

The average age at the time of spinal cord injury (SCI) has increased over time. In the 1970s, the average age at the time of SCI was 28 years old (yo) but as of 2015 has increased to 43 years old (NSCISC, 2022). Despite the typical clinical SCI demographic as that of a middle-aged male, pre-clinical animal models predominately utilize young adult female rodents (Stewart et al., 2020). Recently, we comprehensively reviewed the effect of sex differences in SCI modeling and the importance of including sex as a biological variable (Stewart et al., 2020). Collectively, it is important to consider the significant dichotomy between clinical populations and animal models in the interpretation and applicability of pre-clinical SCI findings in the use toward clinical translation.

In this review, we will discuss why older age at the time of SCI is associated with worse functional outcomes in animal models as well as the confounding variables that affect the interpretation of age-dependent effects clinically. We will review several biological underpinnings of secondary injury and recovery that are affected by the aging process. Specifically, we will cover known physiological aging adaptations that influence SCI responses, exacerbate secondary injury, and worsen functional outcomes. We will also discuss the somewhat unpredictable and unexpected results of animal studies focusing on interventions tailored to treat age-associated physiological differences. The conclusion of our comprehensive evaluation, namely that age can have profound effects on treatment approaches, supports the re-evaluation of pre-clinical therapeutic strategies as well as indicates that the minimal information necessary to translate preclinical results into clinical trials should be reconsidered. Articles in this review were chosen based on a comprehensive review of pre-clinical animal literature covering comparisons between animal models of young adult ages and older, as well as selected clinical reports offering contrasting findings about the role of age in the SCI population.

## Age at time of spinal cord injury and the clinical population

Determining how age at the time of injury affects clinical outcomes after SCI is challenging. Mortality after SCI increases with age creating a potential selection bias where more resilient, or less severely injured, older individuals are a larger representation within longitudinal clinical data (Furlan and Fehlings, 2009). This bias leads to caveats regarding directly comparing across age groups. Additionally, the causes and mechanisms of injury differ between young and older persons, presenting a further confound. While comparing injury responses across age groups in animal models can address some caveats present in clinical data, central cord syndrome (CCS) is a common mechanism of SCI with a higher representation in older persons and is difficult to model in animals. Displacement injuries produced by vertebral distraction can manifest in a pathology similar to CCS (Chen et al., 2016), however, this model of SCI has not been evaluated across different ages in rodents. Overall, CCS is not represented as a common model of SCI in animals. Additionally, animal models of SCI do not include comorbidities commonly found in an aging population such as cardiovascular disease, cancer, etc., which increases the frailty and worsens outcomes of older populations by increasing the frequency of adverse events and length of hospitalization (Velanovich et al., 2013; Banaszek et al., 2020; Dicpinigaitis et al., 2022). Correspondingly, frailty has been correlated as a predictor of mortality in elderly individuals (Carlile et al., 2022). Evidence from thoracoabdominal aortic aneurysm repair indicates that paraplegia risk may be correlated with frailty [using sarcopenia (core muscle loss) as a marker of frailty], however, the extent to which age and frailty interact to affect SCI outcomes remains understudied.

As previously noted, the average age of SCI in the US has increased to 43 years old. The causes, spinal levels, and severity of SCI have also changed over time with the most frequent category of neurological injury being incomplete tetraplegia (48.6% in the US since 2015) (NSCISC, 2022). In the US, the leading cause of SCI across all age populations between 2015 and 2021 was motor vehicle accidents (37.7%), with falls as the second leading cause (31.4%) (NSCISC, 2022). In persons greater than 45 years old, falls are the primary cause of SCI with similar findings in other countries (Toda et al., 2018; NSCISC, 2020; Sun and Zhang, 2021). Particularly in older persons, low speed/low impact falls (from standing) can result in the most common type of incomplete SCI, CCS. Although there is no clear, universally agreed-upon definition of CCS, the clinical presentation includes a greater loss of function in the arms and hands, relative to the lower extremities. Although CCS occurs in younger persons due to high energy impact injuries, in older persons this type of injury is caused by cervical hyperextension from a fall where pre-existing cervical stenosis is present (contributing to spinal cord compression) and is not always associated with spine fracture or dislocation (Avila and Hurlbert, 2021; Ameer et al., 2022). CCS has traditionally been considered to have a higher rate of recovery than other types of SCI, however, a recent publication where individuals with CCS were matched with non-CCS incomplete SCI (by severity and neurological level of injury, age), demonstrated that CCS individuals had less recovery compared with incomplete SCI (Blasetti et al., 2020). This complicates our understanding of how age impacts recovery following SCI, as a high percentage of clinical SCI are cervical, incomplete, with a frequent presentation of CCS, for which there is no animal model. It is anticipated that the incidence of incomplete SCI will increase over time due to trends in age and cause of SCI (Devivo, 2012). This demographic shift will likely include a commensurate increase in CCS, in an active, aging population, presenting a potential confound in the translation of preclinical animal studies to human SCI.

The difficulty in directly comparing outcomes between age groups in humans likely contributes to conflicting reports across retrospective studies examining age at the time of SCI (Scivoletto et al., 2003; Furlan and Fehlings, 2009; Furlan, 2021). For example, Furlan (2021) identified no significant differences in function between older and younger populations 1-year post-SCI from a re-evaluation of data from the first North American Spinal Cord Injury Consortium (NASCIC) trials on methylprednisolone (Bracken et al., 1985; Furlan, 2021). In contrast, several other independent reports have identified worse motor and sensory outcomes in individuals injured later in life (Cifu et al., 1999a,b; Dai, 2001; Seel et al., 2001).

Findings from the Furlan (2021) NASCIC re-assessment examined data from 306 participants treated with methylprednisolone, including 39 females and 267 males with an average age of 31 years old at the time of SCI. The mean age of 31 at the time of SCI, published in 1985, pre-dates the progressive shift toward older age at the time of SCI, which is now approximately 43 years old (Bracken et al., 1985; NSCISC, 2022). In the retrospective assessment, older age was defined as 65 years old or older at the time of SCI. Functional outcomes were determined using the change in NASCIS motor scores [14 muscles assessed on a 6-grade scale ranging from 1 (normal function) to 6 (no contraction) (Bracken et al., 1985)] obtained at 1-year post-injury from scores obtained at the time of admission. Neurological recovery scores were adjusted for confounders of sex, injury mechanism, ethnicity, level of SCI, type of wound (open or closed), consciousness on admission, and dose of methylprednisolone using multiple regression analysis against age at the time of injury. Importantly, in this report, there were only 13 individuals out of 306 participants in the 65 years or older group (Furlan, 2021).

Furlan (2021) identified a significant positive correlation between older age at the time of SCI and improved motor scores at 1-year post-injury. While at first, this appears to contradict pre-clinical studies that find worse outcomes with older age (Gwak et al., 2004a; Genovese et al., 2006; Siegenthaler et al., 2008a,b; Fenn et al., 2014; Hooshmand et al., 2014; Zhang et al., 2015, 2016, 2019; Takano et al., 2017; von Leden et al., 2017; Martín-López et al., 2021; Stewart et al., 2021b), there are several important caveats to consider. First, it is interesting to note that the original publication reported a significant increase in mortality within 1-year of SCI among individuals 50 years or older at the time of injury (Bracken et al., 1985). This significantly increased mortality could have introduced a selection bias preferencing individuals with more robust recovery from injury since data on aged individuals who died was not included when determining motor improvements at 1 year. Next, a trend toward different injury mechanisms in older individuals may indicate less severe injuries at the time of SCI (Furlan et al., 2010). It should be noted, however, that in the retrospective study injury severity scores (Frankel Grade scores) were not significantly different at baseline between young and aged groups (Furlan, 2021). Finally, and what has the most potential relevance to the discussion below, is the potential for methylprednisolone to have exerted an age-dependent effect, conferring larger therapeutic benefit to older individuals. Due to ethical concerns regarding withholding methylprednisolone treatment during the NASCIC study, all individuals enrolled in the study received treatment, no placebo treatment was given (Bracken et al., 1985). Considering data were normalized to baseline, observing an increase in motor recovery relative to baseline with older age might be explained by age-dependent differences in treatment efficacy (Furlan, 2021).

In contrast to Furlan (2021), several other reports associate older age at the time of SCI with worse clinical outcomes as measured by the American Spinal Injury Association (ASIA) Impairment Scale (AIS), functional independence measures, and/or capacity for over-ground locomotion (Bravo et al., 1996; Cifu et al., 1999a,b; Dai, 2001; Seel et al., 2001; Coleman and Geisler, 2004; Wilson et al., 2014; Oleson et al., 2016; Brouwers et al., 2020; Engel-Haber et al., 2020). A more recent meta-analysis of clinical reports collectively identified age as a significant variable associated with worse neurological and functional recovery (Kirshblum et al., 2021). Previously, Seel et al. (2001) reported that rehabilitation performance measures were worse with older age, often requiring increased lengths of stay prior to hospital discharge. Part of the cause of this functional disparity between ages may be due to reduced muscular strength, independent of SCI, in the aging population; a consideration for functional disparities after SCI regarding both age and sex (Thomas and Grumbles, 2014). Age-associated functional outcomes are also strongest after incomplete injuries characterized as AIS B or C (Kirshblum et al., 2021), implicating injury severity as a potential age-dependent caveat. It is important to note that unlike Furlan (2021), not all studies control for confounding variables such as injury mechanism, baseline score, etc. (Dai, 2001; Furlan, 2021). Further, several publications, after adjusting for these confounding variables, have not found age to be associated with worse outcomes or reported weak relationships with age at the time of SCI and functional recovery (Furlan et al., 2010; Wilson et al., 2014; Furlan, 2021). Collectively, age does appear to be associated with worse outcomes, but whether reduced neurological recovery is a product of changing biological responses with aging or differences in clinical scenarios cannot be extrapolated from clinical reports.

## Aging in animal models

### Locomotor and sensory outcome differences

Many caveats with interpreting clinical reports (i.e., mortality, injury type) can be addressed through controlled modeling in animals. Comparing between younger and older rodents in pre-clinical SCI models has elucidated several underlying differences occurring with advancing age in the pathology and recovery following SCI. Specifically, when injury severity, anatomical location, and injury type are controlled across age groups in rats and mice, older age is associated with worse functional outcomes, even when comparing young adults (3–4 months old) to middle-aged (12–14 month-old) rodents (Gwak et al., 2004a; Genovese et al., 2006; Siegenthaler et al., 2008a,b; Fenn et al., 2014; Hooshmand et al., 2014; Zhang et al., 2015, 2016, 2019; Takano et al., 2017; von Leden et al., 2017; Martín-López et al., 2021; Stewart et al., 2021b). The ability to provide immediate and sustained care to reduce age-associated mortality and limit selection bias in animals may be a potential factor in animal studies. Further, animal studies are not confounded by age-dependent differences in central cord syndrome, which is not examined in most rodent models. Indeed, the age-dependent recovery observed in animal models is inconsistent with some clinical reports (Furlan and Fehlings, 2009; Furlan et al., 2010; Furlan, 2021).

An age-associated decrease in locomotor outcomes has been replicated across labs and in both mice and rats. In this review, we will not discuss differences between neonatal/juvenile/pediatric and adult ages but will limit discussion to differences between young-adult, middle-aged, and elderly groups. The majority of reports from rodent studies examining the effects of age at the time of SCI reproducibly demonstrate that older age results in worse functional outcomes. In rats, age-associated impairments in functional recovery have been demonstrated using the Basso, Beattie, Bresnahan scale of locomotor recovery (BBB) (Basso et al., 1995; Gwak et al., 2004a,b; Genovese et al., 2006; Siegenthaler et al., 2008a,b; Hooshmand et al., 2014; Roozbehi et al., 2015; von Leden et al., 2017; Martín-López et al., 2021). Similar results are observed in mice utilizing the Basso Mouse Scale (BMS) (Basso et al., 2006; Kumamaru et al., 2012; Fenn et al., 2014; Zhang et al., 2015, 2019; Takano et al., 2017; Stewart et al., 2021b). To date, only three reports failed to detect differences across older age (Nishi et al., 2020; Hook et al., 2022; Stewart et al., 2022b), one of which utilized immunodeficient Rag2gamma(C) knockout mice in cervical SCI, the implications for which will be discussed in more detail below.

In addition to locomotor recovery, older age at the time of SCI is associated with differences in sensory function in rodents (Gwak et al., 2004b; Gaudet et al., 2021; Stewart et al., 2022b). Use of the Hargreave’s test for thermal hypersensitivity revealed that absolute values for paw withdrawal latency do not differ between ages after SCI, however, there is a pre-existing hypersensitivity with between older mice between 2- and 20-months of age, making the change from uninjured conditions larger in younger mice; importantly this finding was observed in a second independent report between mice of 4- and 14-month of age (Gaudet et al., 2021; Stewart et al., 2022b). In contrast to thermal allodynia, mechanical hypersensitivity is not different between 2- and 20-month mice at baseline but younger mice exhibit a greater sensitivity at 1-week post-SCI which then resolves and plateaus at approximately the same sensitivity as 20-month SCI-mice (Gaudet et al., 2021). While both evoked thermal and mechanical hypersensitivity appear to indicate that younger age is associated with larger changes in hypersensitivity responses after SCI, Gaudet et al. (2021) also reported that 20-month mice exhibit a greater frequency of behaviors associated with spontaneous pain development. Self-severing, or autotomy, occurred at a significantly greater frequency in 20-month, compared to 2-month, mice (Gaudet et al., 2021). Collective results from both mechanical and thermal sensitivity tests suggest that younger, rather than older, mice experience larger changes in hypersensitivity after SCI related to exogenously evoked stimuli, while older mice exhibit a greater frequency of behaviors associated with spontaneous pain (Gaudet et al., 2021). Importantly, an earlier report identified that younger adult mice, but not middle-aged mice, develop mechanical hypersensitivity following spinal hemisection, validating that spinal mechanisms persisting in younger mice may result in a greater chance of developing SCI-induced pain (Gwak et al., 2004b). These findings may be consistent with age-dependent differences in the capacity for axon growth, plasticity, and maladaptive plasticity, which may favor a larger response at younger ages.

### Plasticity and regeneration

Several studies identify that functional recovery is indeed reduced in older rodents after SCI, however, the biological mechanisms that underlie decreased recovery continue to be explored. The aging central nervous system is well-known to possess a decreased capacity for plasticity and regeneration, particularly in the context of hippocampal memory formation (Isaev et al., 2019). Hippocampal neurogenesis declines with age (Ahlenius et al., 2009). A decreased growth potential of mature neurons has also been identified after peripheral nerve axotomy with the speed of axon growth and the total abundance of regenerating axons declining with advanced age (Pestronk et al., 1980; Verdú et al., 2000; Kaneko et al., 2021; Wagstaff et al., 2021). A reduction in trophic factor and cytokine secretion, as well as mitigated intrinsic growth responses contribute to a decreased regenerative potential of peripheral nerves with aging (Stratton et al., 2020; Kaneko et al., 2021; Wagstaff et al., 2021). The influence of aging on axonal repair in the central nervous system has been recently reviewed (Sutherland and Geoffroy, 2020) and will only be discussed briefly below.

To date, there have only been three published reports evaluating the effects of advanced age on regeneration after SCI, with all three reports providing corroborating evidence for observations in other neurological conditions (Jaerve et al., 2011; Roozbehi et al., 2015; Geoffroy et al., 2016). Specifically, all three studies found a decrease in axon growth with older age (Jaerve et al., 2011; Roozbehi et al., 2015; Geoffroy et al., 2016). Results after controlled thoracic transection provided the first evidence that age-dependent decreases in plasticity play a major role in functional recovery after SCI (Gwak et al., 2004a). Axon plasticity and growth after SCI can include both shorter-distance sprouting and long-distance regeneration (Cafferty et al., 2008). There is an emerging understanding that different mechanisms mediate long-distance regeneration relative to short-distance sprouting (Geoffroy and Zheng, 2014). Age-dependent declines in axonal growth have since been observed in SCI conditions with and without interventions aiming to enhance regeneration and sprouting. Both sprouting below the lesion and regeneration of damaged axons are reduced with older age (Jaerve et al., 2011; Roozbehi et al., 2015; Geoffroy et al., 2016).

Exploring the age-dependent effects on different mechanisms of axon plasticity provides insight into the role of age on SCI responses. For example, converging literature identifies intracellular signaling through the mTor pathway as essential for inducing long-distance axon regeneration (Park et al., 2008; Liu et al., 2010; Danilov and Steward, 2015; Du et al., 2015; Geoffroy et al., 2015). One strategy aimed at enhancing mTor activity is through administering the chemokine stromal-derived factor-1 (SDF-1), which acts on g-protein-coupled receptors, CXCR4 and CXCR7 (Opatz et al., 2009). SDF-1 activates the PI3K/AKT pathway and leads to mTOR activation (Dillenburg-Pilla et al., 2015). SDF-1 causes potent axon growth *in vivo* after both optic nerve crush and SCI (Jaerve et al., 2011, 2012a; Heskamp et al., 2013; Negro et al., 2017; Stewart et al., 2017; Li et al., 2021). Jaerve et al. (2011) used SDF-1 to examine how aging differentially affects the potential for sprouting and regeneration within the spinal cord after injury (Jaerve et al., 2011). SDF-1 was infused into spinal cords after a dorsal hemisection in young (9–14 weeks old) and older (22–28 months) rats using osmotic pumps (Jaerve et al., 2011). Without treatment, older rats had reduced sprouting of spared serotonergic (5-HT), tyrosine hydroxylase (TH), and CGRP fibers below SCI lesions. Similarly, although treatment with SDF-1 resulted in more sprouting below the lesions of younger rats, there was little effect of SDF-1 on axon sprouting in older rats. In contrast to short-distance sprouting below the lesion, SDF-1 did induce growth and regeneration of damaged 5-HT and TH fibers into the lesion with no detectable differences between ages. Corticospinal tract (CST) fibers did not grow into or beyond the lesion but were found to sprout more in younger rats rostral to the lesion in response to treatment (Jaerve et al., 2011). Collectively, these data indicate that axons sustain a comparable ability to regenerate, but not sprout, with older age.

Contrasting findings were reported by Geoffroy et al. (2016) utilizing a PTEN knockout model to increase mTor activity and induce axon growth of the CST (Geoffroy et al., 2016). Geoffroy et al. (2016) knocked out PTEN from corticospinal tract neurons at either P1, 4–6-week, 10–week, or 12–18 months of age. They then performed T8 dorsal hemi-sections 4–6 weeks later and evaluated CST regeneration at 6-week post-injury. Increased age at the time of injury blunted axon regeneration caudal to the lesion with 12–18-month mice exhibiting no significant regeneration beyond the injury site. In contrast, younger mice receiving PTEN KO at P1 or at 4–6-week of age, then subsequently injured 4–6 weeks later, had growth and regeneration caudal to the lesion. Geoffroy et al. (2016) replicated an age-dependent decrease in regeneration by evaluating effects of PTEN KO on a second spinal tract, the rubrospinal tract, which is believed to have greater regenerative potential (Geoffroy et al., 2016). Specifically, in this experiment, 6-week-old and 8-month-old mice demonstrated some regeneration caudal to the lesion after PTEN KO, with 6-week-old mice having significantly more regenerating fibers caudal to the lesion and at further distances away from glial boundaries (Geoffroy et al., 2016).

While both SDF-1 and PTEN knockout act to enhance the intracellular mTor signaling pathway, mechanisms of action differ between the two manipulations in the timing, duration, and intensity of the effect. Specifically, a permanent PTEN knockout likely induces a more sustained and intense growth response, evidenced by the magnitude of regeneration across the lesion, and may be more sensitive at detecting an age-dependent decline in axon growth. Further, knocking out PTEN exhibits an effect at the level of the soma which might exhibit a stronger transcriptional effect compared to local infusion of SDF-1 near the lesion. Alternatively, the discrepancy regarding an age-dependent decline in regeneration between studies could be explained either by species differences or in the assessment of different fiber tracts. While the exact extent of how species differences affect regenerative potential is unknown, mice do exhibit a dense collagenous glial scar within the lesion center compared to rats which form cystic cavitation and leave empty fluid filled spaces that are barriers to axon growth. Differences in the scaring response suggests there may be critical differences in the microenvironment affecting the potential for axon growth and regeneration in a species-dependent manner.

The two studies also differed in the methods and fiber tracks analyzed. Specifically, Jaerve et al. (2011) evaluated fibers using immunohistochemical labeling, specifically being 5-HT, TH, and CGRP axons, while Geoffroy et al. (2016) evaluated motor neuron tracts requiring tract tracing, specifically the rubrospinal and corticospinal tracts. While Jaerve et al. (2011) did trace for corticospinal tract growth they were unable to detect a significant regenerative effect into or beyond the lesions, prohibiting analysis of regeneration of this fiber tract. Regardless, both experiments provide evidence that axon growth and plasticity are diminished with advancing age and are less receptive to treatment approaches. Finally, it should be noted that axotomized motor neurons within the cortex of young and aged rats also display diverse transcriptional profiles after SCI which likely plays a role in the different growth responses to injury and intervention (Jaerve et al., 2012b).

### Injury and inflammation

Spinal cord injury causes a robust intraspinal inflammatory response consisting primarily of neutrophils, microglia, and macrophages within the first week of injury. At later timepoints, adaptive immune cells, e.g., b- and t-cells, infiltrate the injured spinal cord. Fenn and colleagues were among the first to provide evidence that older age at the time of injury (3-month vs. 18-month) leads to an exacerbated intraspinal inflammatory response. Specifically, we observed a loss of IL-4 receptor (IL-4R) on microglia and macrophages in 18-month-old mice after SCI (Fenn et al., 2014). IL-4R signaling induces an alternative, anti-inflammatory, macrophage phenotype that enhances tissue repair and regeneration *in vivo* after SCI (Kigerl et al., 2009; Gensel and Zhang, 2015). We observed an age-associated shift of microglia and macrophages toward a more pro-inflammatory phenotype with advancing age that contributed to an exacerbated secondary injury response (Fenn et al., 2014). Subsequently, we observed that older mice (4- vs. 14-month) have an imbalance in inflammatory cytokines surrounding the lesion, favoring a more pro-inflammatory (vs. reparative) environment with advanced age (Zhang et al., 2015). The pro-inflammatory cytokine, IL-12, and anti-inflammatory cytokine, IL-10, are expressed in relatively equal proportions intraspinally in older (14-month-old) SCI mice. In contrast, in young mice (4-month-old), IL-10 expression levels significantly increase over time and protein levels of IL-10 significantly increase more in young vs. aged mice by 7 dpi within the lesion (Zhang et al., 2015).

Older age at the time of injury is also associated with increased recruitment of macrophages into the lesion in both rats and mice (Hooshmand et al., 2014; Zhang et al., 2019; Stewart et al., 2021a). We also observed that intraspinal macrophages in 14-month mice produce significantly less anti-inflammatory IL-10 and significantly more reactive oxygen species (ROS) during the sub-acute stages of SCI relative to macrophages in 4-month animals (Zhang et al., 2015, 2016, 2019). Indeed, older mice have larger lesions and accumulate more oxidative stress by 7-days following T9 contusion SCI (Zhang et al., 2015, 2016). Age-dependent increases in ROS production are attributed to phagocytic cells from older animals expressing higher abundances of NADPH Oxidase 2 (Nox2), which generates the reactive oxygen species, superoxide, in macrophages and microglia (Zhang et al., 2016; Stewart et al., 2021a). Increases in Nox2 with age occurs in both mice and rats and in both traumatic and non-traumatic SCI (Zhang et al., 2016; von Leden et al., 2017; Michaels et al., 2020; Stewart et al., 2021a). An accumulation of ROS end products indicative of oxidative damage, 4-hydroxynonenal (4-HNE), and 3-Nitrotyrosine (3-NT), are increased in older mice at 7-days after SCI (Zhang et al., 2016, 2019; Stewart et al., 2021b), consistent with age-dependent changes in macrophage activation and phenotype. When we targeted the age-dependent increase in Nox2 using apocynin, a Nox inhibitor, we detected a larger therapeutic response in middle-aged (14-month) compared to adolescent (4-month) mice after SCI (Zhang et al., 2019). Specifically, apocynin decreased oxidative stress and intraspinal inflammation in an age-dependent manner and improved locomotor outcomes only in 14-month mice. These age-dependent inflammatory responses have therapeutic implications for SCI and further demonstrate that both the underlying biology of SCI as well as treatment efficacy change with age.

Two other recently published manuscripts provide more evidence highlighting the importance of inflammation in age-dependent effects after SCI. First, Nishi et al. (2020) evaluated how age and mouse strain affect functional outcomes after SCI (Nishi et al., 2020). While the same lab had previously reported an age-dependent decline in recovery in 18-month compared to 3-month rats after cervical SCI (Hooshmand et al., 2014), recovery was not affected after cervical SCI in 4- vs. 16-month Rag2gamma(C) knockout mice (Nishi et al., 2020). Rag2gamma(C) knockout mice are immunodeficient in elements of adaptive immunity, specifically having a loss of T-, B-, and natural killer cells (Nishi et al., 2020), as well as reduced serum IgG (Lee et al., 2018). While the role of infiltrating IgG in the spinal cord acutely after SCI is not thoroughly understood, it likely plays a role in binding to cellular debris to encourage phagocytosis from macrophages (Kopper and Gensel, 2018). Of interest, IgG increases within the spinal cords of wild-type mice in an age- and sex-dependent manner after SCI (Stewart et al., 2022a). Accordingly, Nishi and colleagues mention that the lack of adaptive immune cells may have masked the age-dependent pathophysiology and advocate for examinations of immune cell-age interactions in SCI (Nishi et al., 2020). The lack of age-associated effects after SCI in immunodeficient mice further implicates inflammation as a key regulator of age-associated SCI pathophysiology.

Targeting inflammation by knockout out micro-RNA-155 (miR-155) has also been used as a strategy to determine the effects of inflammatory signaling on pain development in older age (2- vs. 20-month) animals after SCI (Gaudet et al., 2021). miR-155 has been shown to regulate both neuron growth and extension *in vivo* after SCI, as well as attenuate pro-inflammatory signaling in macrophages and mitigate macrophage accumulation within SCI lesions (Gaudet et al., 2016). miR-155 knockout (KO) mice injured at 2 months of age demonstrate an alleviation of hypersensitivity within the first 2 weeks of SCI, whereas miR-155 KO mice injured at 20 months of age do not differ from wild-type controls. In contrast, miR-155 KO did mitigate an age-dependent increase in mortality after SCI as well as an age-dependent development of spontaneous pain. Because miR-155 KO also reduces lesion sizes in adolescent mice (Gaudet et al., 2016), mitigation of pain development might not be exclusively associated with inflammatory modulation and could be attributed to sparing of axons that control pain perception (Gaudet et al., 2021). Regardless, miR-155 KO demonstrates that immune-associated strategies aimed at mitigating pain can also display age-dependent effects (Gaudet et al., 2021). The collective evidence from several reports now implicates age-related changes occurring throughout the inflammatory axis as maladaptive and exacerbate the pathophysiology of SCI.

### Mitochondrial function

Redox metabolism is known to change with advanced age and resembles a shift toward a stronger reliance on glycolysis for energy production, a term coined the Warburg effect which was originally identified in cancer (Samudio et al., 2009; Burns and Manda, 2017) and is emerging as a hallmark of age-related differences in SCI (von Leden et al., 2019). Mitochondria utilize proton gradients built up within the inner membranous space to drive ATP production. Mitochondria are said to be coupled when the electron transport chain produces a proton gradient in the inner membranous space and when that gradient increase necessitates an increase in ATP production. When the mitochondrial membranes are permeable to protons, the gradient dissipates and electron transport fails to increase ATP production, which is termed an uncoupled response (Berry et al., 2018). In an uncoupled mitochondrion, protons produced from the electron transport chain do not contribute to ATP production. Mitochondria regulate this proton gradient through changing cellular respiratory rates and/or activating mitochondrial uncoupling proteins which allow protons to flow back into the mitochondrial matrix from the inner membrane space (De Simone et al., 2015; Zhao et al., 2019).

While at first it may seem maladaptive to intentionally uncouple mitochondria due to the suppressive effects on ATP production, a proton gradient that is too strong induces resistance to electron flow through the respiratory membrane complexes and generates ROS in the form of superoxide (Berry et al., 2018). When the resistance of electron transfer increases, electrons are captured by electrophilic soluble oxygen to form superoxide instead of reducing their energy state through the electron transport chain to form CO_2_ (Berry et al., 2018).

Physiological coupling of mitochondria is regulated on a spectrum to mitigate and control free-radical formation at the balance of maintaining cellular energy demands. Older mitochondria exist in a more uncoupled state relative to younger mice, and consequently, produce less ATP for cellular energy (Conley et al., 2007; Chistiakov et al., 2014; Stefanatos and Sanz, 2018). Paradoxically, despite observing mild uncoupling with older age at rest, older mitochondria generate more ROS, which likely underlies the sustained mild uncoupling effect (Stefanatos and Sanz, 2018). Observing an increase in ROS production despite being more uncoupled suggests that underlying dysfunction accrues in mitochondria with advanced age. Indeed, older mitochondria are less capable of buffering cytosolic calcium before opening the mitochondrial permeability transition pore (MPTP) which is known to activate caspase cascades and initiate apoptosis (Mather and Rottenberg, 2000; Paradies et al., 2013). These findings suggest that older mice are more susceptible to both an increase in mitochondrial ROS production and exhibit decreased capacity for buffering cytosolic calcium before opening the MPTP, of which increased intracellular calcium is known to participate in excitotoxicity and secondary injury after SCI.

**FIGURE 1:**
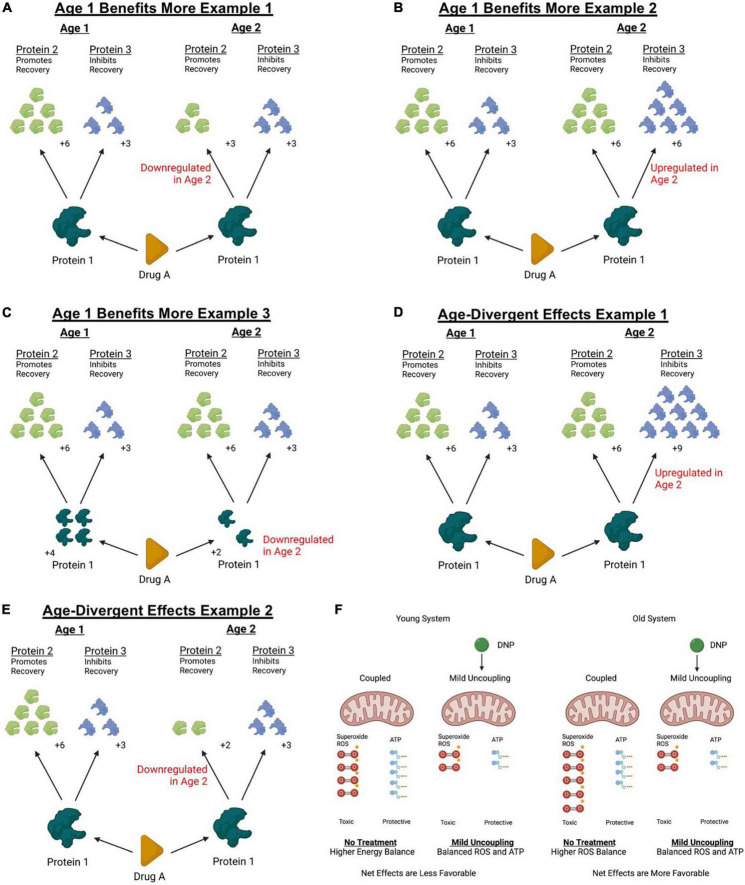
Theoretical models of how age can affect response to treatment. Treatments targeting molecular mechanisms often exert downstream influence on several biological targets. Downstream effectors of a therapeutic intervention can exert both permissive and detrimental effects that can compete to determine the net outcome of a treatment. Aging can change protein abundances of diverse molecular pathways as well as affect cellular responses to injury. Panels **(A–F)** represent some simplified theoretical models of how changes occurring with age can affect net responses to treatment. **(A)** For example, if a reparative downstream effector of an interventions biological target is downregulated with age, older systems might not respond with as large of a therapeutic response. **(B)** Similarly, if a detrimental effector of the intervention is upregulated with age, the net effects might appear smaller in an older animal or disappear altogether. **(C)** Further, the abundance of the biological target itself could be differentially regulated with age and affect treatment efficacy. **(D)** It could also be possible for the net balance of a treatment to change from a beneficial effect to a toxic effect if detrimental downstream regulators are upregulated beyond the effectors that might confer a treatment benefit. **(E)** Or finally, the abundance of the beneficial biological target could be downregulated with age to a point where detrimental effects outweigh a therapeutic effect. In the case of a discussed example above (see Section “Mitochondrial function”), treating 4- and 14-month mice with a mild mitochondrial uncoupler exerted age-divergent effects. **(F)** While it is unknown exactly why the age divergent response was observed, it could be possible that the duality of the treatment response shifted toward conferring a beneficial outcome in older mice. Knowing that older mitochondria produce more ROS and less ATP at rest, it remains possible that older mice exhibited a greater decrease in ROS sufficient to outweigh the detrimental effects of reduced ATP production while uncoupled. This figure was created with BioRender.com.

*In vivo* delivery of small doses of the pharmacological uncoupler, 2,4-dinitrophenol (DNP) can induce a mild-uncoupling response that aids in reducing ROS produced by mitochondria (Jin et al., 2004; Pandya et al., 2007; Patel et al., 2009; da Costa et al., 2010; Geisler et al., 2016; Stewart et al., 2021b; Figure 1F). Mitochondrial ROS production is believed to be a major source of free-radical damage after neurotrauma and treating mice and rats with DNP to mildly uncouple mitochondria is neuroprotective in rats with SCI and TBI as well as mice with TBI (Jin et al., 2004; Pandya et al., 2007; Patel et al., 2009). Our first report using DNP to treat SCI in mice, rather than rats, indicated toxic effects on younger mice and therapeutic effects on middle-aged mice (Stewart et al., 2021b). Specifically, three separate experiments identified that a very mild dose of DNP (1.0 mg/kg/day) exerted reciprocal effects between younger (4-month) and older (14-month) mice after SCI in several outcomes including locomotor abilities, tissue sparing, and mitochondrial function (Stewart et al., 2021b). Because DNP not only mitigates ROS production, but also lowers calcium buffering by mitochondria (Tsai et al., 1997; Korde et al., 2005), there are several potential reasons that could account for an age-divergent response to uncoupling (Stewart et al., 2021b). Regardless, the important take-home message is that a similar dose of the same drug, DNP in this experiment, had opposite effects on outcomes that were dependent upon age at the time of SCI (Stewart et al., 2021b). This study has provided a profound example of why including age as a biological variable either prior to clinical trials or in analyzing findings from clinical trials is important for defining populations that may be sensitive to treatment effects.

### Redox metabolism

Mitigating free-radical damage has been an emphasis for treating acute SCI for several decades (Braughler and Hall, 1982, 1983; Anderson et al., 1985; Bracken et al., 1997). Indeed, the anti-inflammatory and antioxidant effects of methylprednisolone established a rich history of improving outcomes in animal models of SCI, and to a more controversial extent, in humans (Anderson et al., 1985; Bracken et al., 1997; Hall, 2016). Numerous studies identify increases in ROS production and subsequent damage with older age at the time of SCI in rodents (Genovese et al., 2006; Zhang et al., 2016, 2019; von Leden et al., 2017; Stewart et al., 2021b, 2022b). Several techniques have been used to better understand ROS accumulation with older age. First, immunological labels against downstream products of ROS damage, 4-HNE and 3-NT, are upregulated with age at 7-dpi in mouse spinal cord sections and homogenates (Zhang et al., 2016; Stewart et al., 2021b) and up to 30-dpi in rat spinal cord homogenates (von Leden et al., 2017). While evidence of an age-dependent ROS accrual is found at 7-dpi in mice, neither 4-HNE or 3-NT are upregulated in an age-dependent manner at 3-dpi (Zhang et al., 2016; Stewart et al., 2022b). The observation of delayed age-dependent oxidative damage between 3- and 7-dpi implicates infiltrating macrophages as a facilitator of ROS damage with older age (Zhang et al., 2019). After SCI, macrophages emerge at 3-dpi, peak between 7- and 14-dpi, and persist chronically after injury (Beck et al., 2010).

In addition to immunological labeling to detect oxidative damage, systemic delivery of dihydroethidium (DHE), a dye sensitive to superoxide formation, reveals an age-dependent increase in active ROS production at both 3- and 7-dpi (Zhang et al., 2016). DHE is a fluorescent dye that undergoes a red spectral shift upon reacting with superoxide and differs from immunological labeling by being an indicator of active, ongoing, ROS production rather than an accumulation of end products (Peshavariya et al., 2007). Using DHE, we revealed that the largest percentage of cells in the spinal cord oxidizing DHE at 3-dpi were macrophages or microglia (Zhang et al., 2016). This observation is consistent with our subsequent observations of an age-dependent increase in Nox2 within macrophages and microglia (Zhang et al., 2016, 2019; Stewart et al., 2021a) and strengthens the argument that an age-dependent increase in ROS damage is caused by altered macrophage activation. Reports as to whether macrophages infiltrate in greater number after SCI at older ages are inconsistent (Hooshmand et al., 2014; von Leden et al., 2017; Zhang et al., 2019; Furlan et al., 2020; Stewart et al., 2021a; Li et al., 2022), however, all studies evaluating differences in macrophage physiology with age identify that older macrophages present with phenotype characteristics of greater ROS production after SCI (Hooshmand et al., 2014; von Leden et al., 2017; Zhang et al., 2019; Stewart et al., 2021a; Li et al., 2022). Taken together, collective data has identified ROS as an age-dependent contributor to SCI pathophysiology.

While the capacity to defend against free radicals is quite complex, one major cellular pathway, the glutathione (GSH) system, has been investigated for its known changes occurring with age (Jones et al., 2002; Liu, 2002; Ghosh et al., 2014; Ferguson and Bridge, 2016). Cellular GSH regulation utilizes a series of GSH peroxidases (Gpx) to sequester different types of free radicals. GSH is a re-usable tripeptide antioxidant that is used as a substrate by Gpx to reduce radicals and is recycled back into a usable form by GSH reductase at the expense of NADPH. Advanced age diminishes cellular levels of both GSH as well as the availability of its amino-acid constituent, cysteine, within the plasma (Jones et al., 2002; Liu, 2002; Ghosh et al., 2014; Ferguson and Bridge, 2016). The ability to produce and maintain adequate cellular GSH levels is believed to be bottlenecked by either the availability of its cysteine substrate or the availability of the enzyme responsible for the first of two ligation reactions: glutamate-cysteine ligase (GCL) (Griffith, 1999; Lu, 2009). Both GSH and GCL abundance decrease with age in other organ systems (Liu, 2002; Ghosh et al., 2014; Ferguson and Bridge, 2016). Further, both GSH and GCL activity diminishes following SCI. We recently reported that GSH does indeed diminish with older age (4- vs. 14-month mice) within the spinal cord independent of injury, identifying a reduced capacity for older rodents to defend against oxidative stress after SCI (Stewart et al., 2022b).

Glutathione depletion after SCI occurs as early as 24-h post-SCI and remains depleted for up to 72-h, and likely longer, with greater decreases at 72- compared to 24-h post-injury (Patel et al., 2014; Stewart et al., 2022b). Interestingly, however, 14-month mice did not have a consistent decrease in GSH after SCI, likely a consequence of having pre-depleted levels of GSH in uninjured conditions (Stewart et al., 2022b). Gpx activity levels in 4-, but not 14-month, mice is significantly increased in response to SCI (Stewart et al., 2022b). In contrast, 14-month mice have an increase in Gpx activity independent of SCI (Stewart et al., 2022b), likely as a compensatory mechanism responding to increased basal levels of ROS production with advanced age within the spinal cord (von Leden et al., 2017). Taken together, an age-dependent decrease in GSH likely sensitizes older mice to oxidative stress acutely after spinal trauma (Stewart et al., 2022b).

Targeting GSH dysfunction after SCI has been performed by providing cysteine analogs such as n-acetylcysteine (NAC) or a more recently developed *n*-acetylcysteine amide (NACA) which has better bioavailability within the spinal cord (Kamencic et al., 2001; Hanci et al., 2010; Karalija et al., 2012, 2014; Patel et al., 2014; Guo et al., 2015; Olakowska et al., 2017; Stewart et al., 2022b). Treating SCI in rats with NAC or NACA restores cellular levels of GSH, protects against oxidative stress, and improves mitochondrial, behavioral, and histopathological outcomes (Kamencic et al., 2001; Hanci et al., 2010; Karalija et al., 2012, 2014; Patel et al., 2014; Guo et al., 2015; Olakowska et al., 2017). Owing to a pre-existing decrease in GSH with older age, as well as evidence of more ROS damage accumulating in older SCI-mice (Zhang et al., 2016, 2019; Stewart et al., 2021b), we predicted that NACA treatment would have a robust protective effect in 14- compared to 4-month mice. Contrary to this hypothesis, 14-month mice treated with NACA trended toward worse functional and histopathological outcomes, despite observing a significant increase in GSH and an improved redox ratio (GSH/GSSG) (Stewart et al., 2022b). While the mechanisms underlying these trends remain unknown, this work provides yet another example of how treatment responses can be unpredictable at different ages, even when the biological underpinnings change with age in a manner that point to a seemingly obvious outcome.

### Mortality and health differences

While the effects of aging on inflammation, mitochondrial function, and redox metabolism have been well-characterized in many physiological systems, there are other consequential interactions between age and SCI not directly related to central pathology. Similar to clinical findings, older mice experience greater mortality after SCI (Genovese et al., 2006; Takano et al., 2017; Stewart et al., 2020; Gaudet et al., 2021; Martín-López et al., 2021). Findings from several independent laboratories indicate that older mice die more frequently compared to younger mice within weeks following injury (Genovese et al., 2006; Takano et al., 2017; Stewart et al., 2020; Gaudet et al., 2021; Martín-López et al., 2021). More intriguingly, older male mice experience a higher mortality compared to older females at 14-months of age (Stewart et al., 2020). Clinical reports are similar, finding that either advanced age or being male is associated with increased mortality within a year post-SCI (Furlan and Fehlings, 2009; Furlan, 2021). The reasons for increased mortality with age in humans are likely multifaceted and difficult to model in rodents (as discussed in the next paragraph). Even in rodent models where comorbidities can be controlled, the reason for this age- and sex-dependent mortality remains unknown.

The consequences of being older at the time of injury are not just associated with an increase in unexpected death but are also associated with other measures of morbidity such as weight loss. Older rats and mice lose more weight after SCI as a percentage of body weight compared to younger rats and mice (Siegenthaler et al., 2008b; Stewart et al., 2020). Notably, weight changes in animal models follow a different trajectory compared to clinical populations and may be a better metric of morbidity in animal models. Both weight loss and gain are observed in clinical populations depending on several factors potentially affected by mobility and rehabilitation (Powell et al., 2017). Where-as total weight loss may not significantly differ between ages after SCI in humans (Powell et al., 2017), older age has been associated with a shift toward less lean muscle mass and more body fat distribution (Spungen et al., 2003).

The cause of increased mortality in older male mice remains unknown, but our observations point to a potential hematologic contributor. We recently reported that 14-month male mice appeared colder to the touch in the days following SCI and that 14-month male mice had noticeable less red blood cells (RBCs) in the blood after spinning down plasma compared to 4-month mice (Stewart et al., 2020). SCI induced a sex-by-age interaction in the RBC/Plasma ratio when normalized to sham-injured controls with ratios significantly decreased in older 14-month male mice by 28-dpi relative to younger 4-month male mice after injury. When evaluating the effects of aging alone on hematopoiesis a few studies have corroborated these findings. Male mice are reported to have a significant decrease in hematopoiesis during middle age whereas females do not (So et al., 2020). Anemia is reported in middle-aged male mice independent of SCI with the effects profound enough that the authors proposed using the aged C57BL/6 male mice as a model for anemia (Guo et al., 2014). Whether this age- and sex-dependent decrease in RBCs is relevant to the observed mortality remains unknown, but does point to a potential systemic contribution to outcome differences that change with age, and most importantly in a sex-dependent manner after SCI.

Finally, a recent study by Hook et al. (2022) identified that bone volumes decrease in an age-dependent manner. Both male and female mice experience a loss of bone volume with aging alone (2–3 vs. 20–23 months), however, only younger mice have a compounding loss of bone volume after SCI. While SCI did not compound an age-dependent loss in bone volumes, Hook et al. (2022) emphasize that the significant loss of bone volumes independent of injury in older mice could already be approaching a floor effect, thus prohibiting the identification of an SCI-induced decrease. An important emphasis in the study by Hook et al. (2022) is that older mice did not present with worse functional outcomes. While only a few reports have not identified an effect of aging on motor outcomes (Nishi et al., 2020; Hook et al., 2022; Stewart et al., 2022b), in the study by Hook et al. (2022), a lack of functional differences presented a unique opportunity to understand that systemic effects of SCI, rather than functional ability/activity or weight supporting, might underly the bone loss at younger ages (Hook et al., 2022).

### Male and females do not age the same

While work including age or sex as a biological variable is amassing, it remains important to recognize that males and females do not biologically age the same. The influence of sex hormones and how they change with age can give rise to reciprocal effects on several biological processes. For example, estradiol, which is believed to exert neuroprotective effects in SCI, sharply declines during menopause in humans (Kachadroka et al., 2010; Koebele and Bimonte-Nelson, 2016; Stewart et al., 2020). Not only is this hormonal trajectory unique to females, but it also does not occur to the same extent in rodents. In contrast to humans, mice, and rats experience only a small decrease in estradiol relative to the average plasma estradiol in younger rodents (approximately 75% of average) (Lu et al., 1979; Dubal et al., 2012), but maintain a chronic retention of estradiol after reproductive senescence (Koebele and Bimonte-Nelson, 2016; Stewart et al., 2020). Young female rodents undergo normal cycling of the estrus cycle and experience a corresponding cycling of plasma estradiol levels (Lu et al., 1979). In contrast, reproductive senescent rodents sustain a chronic estrous phase and corresponds to a sustained maintenance of estradiol in the blood (Lu et al., 1979). Depending on the cycle stage in younger rodents and the age of the older comparison, the sustained estradiol levels in reproductive senescent rodents may be higher or lower compared to younger rodents which increases the challenges with comparing across ages (Lu et al., 1979). It is therefore important to acknowledge that animal models focused on elucidating the effects of aging can be difficult to extrapolate to the human condition, particularly if only female rodents are utilized.

Similar to humans, testosterone levels in rodents decreases with advancing age (Machida et al., 1981). While the role of testosterone on SCI injury and recovery is poorly understood, there is evidence that testosterone may play a neurotoxic role (Hauben et al., 2002). Specifically, castration of mice and rats prior to SCI or delivery of a testosterone antagonist both resulted in improvements in motor recovery in males after SCI. It therefore may be possible for an age-related decline in testosterone to be mildly neuroprotective within the injured spinal cord. In contrast, however, a decline in testosterone with advancing age is associated with reduced erythropoiesis which leads to an increase in anemia in older mice (Guo et al., 2014), and may account for an increased mortality found at older ages in rodents. Ultimately the effects of decreased testosterone with age on the SCI central or peripheral pathology is not well-understood.

Thus far, we have identified several sex-by-age interactions which validate that some age-dependent injury responses are sex-specific. Specifically, the example provided above that identified a sex-by-age interaction of RBC/Plasma ratio is indicative of how aging can differentially affect outcomes in a sex-specific manner (Stewart et al., 2020). Further, we and others have previously reported that male mice have an early acute proliferation of microglia within and surrounding both SCI and TBI lesions (Doran et al., 2019; Stewart et al., 2021a), however, this sex difference disappears in middle-age (Stewart et al., 2021a). Further, IgG which infiltrates the spinal cord following SCI follows an age- and sex-dependency with 14-month female mice having a significantly larger increase with older age compared to male mice (Stewart et al., 2022a). Although additional studies are needed to evaluate sex-by-age interactions in animal models of SCI, these three examples highlight that the effects of aging may not be generalizable across sexes.

### Complications with investigating age as a biological variable in animal models

While a consensus across several labs has concluded that older age reduces functional recovery after SCI in rodents, it remains important to highlight a few potential caveats that exist in animal modeling. First, it is impossible to test the same cohort of mice at two different ages at the same time, resulting in the use of different cohorts to represent differences in age. In some cases, the challenge associated with obtaining older mice has resulted in experimental strategies which utilize either retired breeders from animal colonies or the use of mixed populations of young and old mice from different colonies such as those purchased from the Jackson laboratories, Charles River, and the National Institute of Aging (NIA) aged animal repository. Utilizing animals from different colonies has an inherent potential to influence the aging process and SCI pathophysiology in unanticipated ways. For example, the C57BL/6 mouse lines have developed different but known mutations within the Charles River and Jackson Laboratory colonies. C57BL/6N mice from Charles River carry a recessive mutation in the Crb1 gene known to induce ocular lesions that impair visual perception in homozygous mice from the breeding colony (Mattapallil et al., 2012). In contrast, C57BL/6J mice from the Jackson Laboratory carry a mutation in the nicotinamide (NAD) nucleotide transhydrogenase (NnT) gene which induces impaired glucose metabolism (Ripoll et al., 2012; Gameiro et al., 2013), and affects inflammatory macrophage phenotypes (Ripoll et al., 2012; Fontaine and Davis, 2016). Importantly, however, while the C57BL/6 mouse colony from NIA is maintained by Charles River, the colony is separate from the Charles River mice and originated from the Jackson Laboratory C57BL/6J strain. This provides one example of how matching cohorts appropriately can be challenging in aging studies. Regardless, aging has been a predictor of worse recovery across all age-matching strategies.

Another complication with including different ages is the role of anatomical growth both of the animal as a whole, as well as the spinal cord in particular. Mice and rats gain weight with age, which can confound the interpretation or analysis of results. Of significant interest is an increase in spinal cord size and diameter with aging (Hooshmand et al., 2014; Stewart et al., 2021a,b). Fourteen months mice have a spinal cord of up to 1.4x the diameter of a 4-month mouse which adds challenges to interpreting outcomes. For example, 14-month mice have significantly larger lesions compared to 4-month mice after SCI, but when data is normalized to a percent of total tissue the differences between ages is reduced (Hooshmand et al., 2014). In this situation, the same injury force manifests in a larger total area of lesioned tissue in older mice, but the magnitude of an age-dependent effect is reduced when considering proportional differences in size. The size difference of the spinal cord has the potential to affect other outcomes as well, such as evaluating for inflammatory cells within or surrounding the lesion (Li et al., 2022). For example, event counts obtained from flow cytometry might represent more total tissue obtained from older mice even if the length of tissue obtained was held consistent. Evaluation of cells using stereological approaches to obtain a cell count density per-cubic area would normalize outcomes but could also be compromised by how data is normalized. Normalizing to lesion area might not provide the most meaningful outcomes, particularly if one would not expect cell densities to differ within the immediate lesioned environment. Normalizing cell counts to total section area might provide a better comparative assessment between two age groups as opposed to limiting assessments within the lesion or absolute counts. Regardless of the details, studying age as a biological variable is complicated by nuanced differences that are not readily apparent on a first-glance analysis of data, such as an age-dependent accumulation of auto-fluorescent lipofuscin in macrophages that can affect immunohistochemical analyses (Vida et al., 2017).

Baseline differences are not simply limited to anatomical discrepancies that develop with aging. Baseline differences in functional outcomes, such as sensory thresholds and measures of forelimb strength, are reported to decline with older ages in uninjured rodents (Gaudet et al., 2021; Stewart et al., 2022b). In three separate studies, two evaluating thermal sensitivity and one forelimb strength, older mice had lower baseline values prior to SCI but identical absolute values compared to younger mice after injury (Nishi et al., 2020; Gaudet et al., 2021; Stewart et al., 2022b). If interpreting data as an absolute value, there is no difference between ages in thermal sensitivity of the hind paws after thoracic SCI, or strength of the ipsilateral forepaw after cervical hemi-section. If these values were interpreted as a percent change from baseline, it would appear that younger mice had a larger proportional loss. It becomes difficult to extrapolate these outcomes to clinical relevance when this age-dependent effect was driven by differences at baseline. Specifically, it is difficult to answer whether absolute values or percent change from baseline are the most meaningful outcome to consider.

Finally, while mortality can be mitigated to a degree in animal models, as reported above, several reports have detected an increased early death of mice and rats after SCI at older ages (Genovese et al., 2006; Takano et al., 2017; Stewart et al., 2020; Gaudet et al., 2021; Martín-López et al., 2021). Observing differences in mortality does potentially introduce a selection bias by allowing successful data collection only from the most robust older animals. Overall, while it is possible to control and account for many confounding variables introduced by including age as a biological variable in pre-clinical SCI studies, there are still important considerations for evaluating age and appropriately interpreting obtained data.

## Discussion

To summarize key findings from this literature review, age as a biological variable effects SCI injury and recovery processes as well as responses to treatments in often unpredictable ways. The average age at time of injury has increased to a mean age of 43 which creates a need to better understand the role of age in the SCI pathophysiology. There are several differences in the etiology of SCI between younger and older clinical demographics, the most pronounced of which is the primary mode of injury is caused by a higher prevalence of slip-and-fall accidents at older age. Slip-and-fall accidents are hallmarked by less severe conditions, often manifesting in central cord syndrome-like pathology. Differences in the primary mechanism of injury and/or severity of the initial insult makes comparing outcomes throughout the spectrum of age challenging. Indeed, several clinical reports implicate older age at the time of SCI as a variable which negatively impacts recovery, however after accounting for confounding variables, the impacts of age are less clear. The use of animal models to better understand the recovery potential throughout the spectrum of age has reproducibly determined that older age limits the recovery of motor functions after SCI. Mechanisms underlying a diminished functional recovery after SCI at older ages have implicated a reduced capacity for axon growth and regeneration in both non-intervened and intervened conditions, as well as more severe secondary injury cascades. Aging affects the acute pathophysiology of SCI through changes occurring at the level of inflammation as well as subcellular microenvironments. Macrophages which infiltrate the lesion after SCI display more aggressive and pro-inflammatory phenotypes that generate more reactive oxygen species at older ages. Within the cell, mitochondria accumulate age-related dysfunctions even prior to SCI that result in the generation of more oxidative stress as well as a reduced capacity to buffer cytosolic calcium. Oxidative damage is increased in the sub-acute stages of SCI at older ages, which can be explained likely by a combination of both an increase in the production of reactive oxygen species and a decrease in antioxidant defense. Most intriguingly, treating older mice after SCI with several antioxidant-based strategies has resulted in outcomes which would either be predictive based on the underlying biology, or completely counterintuitive to the logical hypothesis. The examples provided in this review emphasize an emerging pre-clinical notion that age at time of SCI can unpredictably affect treatment responses.

## Conclusion

Using animal models of SCI, we have identified a precedence for considering age as a biological variable in both pre-clinical and clinical research studies aiming to develop treatment approaches. The underlying pathophysiology of SCI changes with age in meaningful ways and treating those biological maladaptation’s yields unpredictable results. We have, thus far, identified treatment approaches that display larger and smaller treatment effects in older animals, as well as opposite effects to those observed in younger rodent counterparts. While we elucidate how aging affects different elements of the pathophysiology of SCI, we are finding that treatment approaches might not act the same throughout the spectrum of age. Most interventions acting on biological targets often have multiple effects, some beneficial to recovery and others detrimental, and changing the balance of these nuanced effects can influence the outcomes of treatment efforts. It is not difficult to imagine models in which a single molecular target can both promote recovery and damage, and to identify how changes in a system with older age can alter the net response to treatment (Figure 1). Following this review, we propose that therapeutic treatments should be examined across the spectrum of age in pre-clinical models prior to investing time and resources into human investigations. Results from animal testing will re-enforce clinical trial design by providing insights into which age ranges are most susceptible to experiencing a benefit from treatment, while simultaneously helping to avoid potential adverse outcomes that can hurt both the clinical trial success as well as individuals. The pre-clinical findings reviewed above make an argument for a need to include age as a biological variable in clinical research design and interpretation, at the very least by using animal models to guide the age inclusivity. Beyond the implications to clinical research, emerging evidence in basic science is supporting the idea that treatment efforts deemed marginal or insignificant in young rodent models may potentially exert a more significant effect at older ages, and therefore can implicate a re-evaluation of treatment efforts in older animal models. Collectively, work highlighted above emphasizes the need to include age as a biological variable for emerging treatment approaches to help more accurately predict safety and efficacy of therapeutic advances toward clinical trials.

## Author contributions

AS and JG: conceptualization and funding acquisition. JG: supervision. LJ: writing of the clinical aspects. All authors: writing and approving the submitted version.

## Abbreviations

3-NT: 3-nitrotyrosine
4-HNE: 4-hydroxy-non-enol
5-HT: serotonin
BBB: Basso Beattie and Bresnahan scale of locomotor recovery
BMS: Basso mouse scale
DHE: dihydroethidium
DNP: 2,4-dinitrophenol
dpi: days post-injury
GCL: gamma glutamyl-cysteine ligase
GPx: glutathione peroxidase
GSH: glutathione
IgG: immunoglobulin G
KO: knockout
MO: months old
MPTP: mitochondrial permeability transition pore
NAC: *N*-acetylcysteine
NACA: *N*-acetylcysteine amide
NASCIC: North American Spinal Cord Injury Consortium
NnT: nicatinamide nucleotide transhydrogenase
NOX: NADPH oxidase
PTEN: phosphatase and tensin homologue protein
RBCs: red blood cells
ROS: reactive oxygen species
SCI: spinal cord injury
SDF-1: stromal derived factor 1
TBI: traumatic brain injury
TH: tyrosine hydroxylase
YO: years old.
